# Unveiling brucellosis risks in breeding herds: Case study on diagnostic failures and control gaps in South Africa

**DOI:** 10.4102/ojvr.v93i1.2264

**Published:** 2026-07-31

**Authors:** Koketso D. Mazwi, Emmanuel P. Lita, Ayesha Hassim, Emmanuel Seakamela, Itumeleng Matle, Farah Abdool-Khader, Gerhardus S. Scheepers, Yusuf B. Ngoshe, Henriette van Heerden

**Affiliations:** 1 Department of Veterinary Tropical Diseases, Faculty of Veterinary Science, University of Pretoria, Pretoria, South Africa; 2 Department of Clinical Microbiology and Infectious Diseases, Faculty of Health Sciences, University of the Witwatersrand, Johannesburg, South Africa; 3 School of Veterinary Medicine, University of Juba, Juba, South Sudan; 4 Bacteriology Division, Agricultural Research Council, Onderstepoort Veterinary Research, Pretoria, South Africa; 5 Department of Agriculture and Animal Health, College of Agriculture and Environmental Sciences, University of South Africa, Florida, South Africa; 6 Gauteng Department of Agriculture and Rural Development, Johannesburg, South Africa; 7 Zodiac Dierekliniek Veterinary Clinic, Brits, South Africa; 8 Department of Production Animal Studies, Faculty of Veterinary Science, University of Pretoria, Pretoria, South Africa

**Keywords:** brucellosis, *Brucella abortus*, cattle, sable antelope, AMOS-PCR

## Abstract

Brucellosis is a zoonotic pathogen of livestock, wildlife and humans, with significant implications for reproductive losses and public health. The movement and introduction of breeding animals with unknown status into previously *Brucella*-negative herds pose a risk for disease introduction and sustained transmission. This case study documents two independent brucellosis outbreaks integrating epidemiological observations with serial serological testing, bacteriological culture and molecular confirmatory assays following abortion events. Diagnostic tests were performed, including serology, PCR and culture from diverse samples. On a cattle farm, animals previously certified as brucellosis-negative demonstrated progressive seroconversion from 2024 onwards. *Brucella abortus* was detected in the blood clots of tested animals using abortus-melitensis-ovis-suis PCR (AMOS-PCR), including a suspect bull that later tested seronegative on follow-up serology. A *Brucella*-positive human case occurred following direct exposure to the infected cattle farm, underscoring the zoonotic risk. On the sable antelope farm, *B. abortus* was detected in two seropositive females through culture of lymph nodes and amniotic fluid, while *B. abortus* was also isolated from the semen of a seronegative breeding bull, suggesting that the bull was a carrier and posing a transmission risk to the herd. The findings demonstrate that infected breeding males may remain seronegative while actively harbouring and shedding *B. abortus*. This study indicates that seronegative breeding males can undermine brucellosis control programmes, may act as undetected reservoirs of infection, thereby challenging current surveillance strategies. The findings highlight the diagnostic limitations of relying solely on serological tests, particularly in chronically infected cattle and wildlife, and support the integration of more sensitive assays, such as iELISA, alongside PCR methods, into diagnostic strategies on brucellosis-affected farms to improve detection and support eradication efforts.

**Contribution:**

Strengthened biosecurity, systematic testing of breeding males, and comprehensive vaccination programmes are critical to preventing ongoing spillover among livestock, wildlife, and humans in South Africa.

## Introduction

Brucellosis is a bacterial zoonotic disease, caused by various species of *Brucella*, primarily affecting domestic and wild animals with humans as accidental hosts (Corbel 2006). It remains a disease of major public health and economic significance, particularly in regions with extensive livestock production (Franc et al. 2018). The control of zoonotic diseases, including brucellosis, in human populations has largely depended on the management of animal diseases, including vaccination (Corbel 1997; Godfroid et al. 2005). For brucellosis, mainly caused by *Brucella abortus*, the most effective vaccine (*B. abortus* S19) is administered solely to calves, as vaccination of adults interferes with serological testing and can lead to reproductive complications, including orchitis and epididymitis in males, prolonged infection, and potential abortion in pregnant females (Lalsiamthara & Lee 2017).

*Brucella abortus* S19 and *Brucella melitensis* Rev 1 are established as effective vaccines for preventing *B. abortus* in cattle, and for protecting against *B. melitensis* and *Brucella ovis* in sheep and goats, respectively (Elberg 1996; Nicoletti 1990). Routine surveillance and control programmes largely focus on female animals, as they are the main source of brucellosis transmission through abortion and milk shedding. National surveillance programmes for bovine brucellosis generally focus on animals with the highest risk of transmitting infection through reproduction or milk, particularly adult females, and breeding stock, while males are tested selectively, particularly when used for breeding, linked to an infected herd, or required for movement compliance (Code of Federal Regulations [CFR] 2025; Khurana et al. 2021; United States Department of Agriculture [USDA] 2022). Although clinical signs such as orchitis and epididymitis may occur in males, serological testing and interpretation have historically been designed for herd-level detection, leading to a testing strategy centred on females and breeding animals rather than routine testing of all males (Godfroid, Nielsen & Saegerman 2010).

Serological testing, including the rose Bengal test (RBT), complement fixation test (CFT), and indirect enzyme-linked immunosorbent assay (iELISA), is therefore commonly targeted at females, both to identify infected individuals and to prevent spread within herds. Targeting females in diagnostic programmes allows for more effective detection of infection and supports vaccination strategies aimed at reducing reproductive losses and zoonotic risk (World Organization for Animal Health [WOAH] 2023). However, heifers born to infected cows often test negative before calving, with seroconversion typically occurring only after the birth of their first calf (Crawford, Huber & Sanders 1986). A major challenge in brucellosis control is diagnosing latent and chronic infections, which often escape detection by serological tests and even culture, particularly in endemic regions, where such infections contribute to underreporting and hinder eradication efforts.

Female animals typically present with abortion, stillbirth, or retained placenta, while males may develop orchitis, epididymitis, and seminal vesiculitis (Corbel 2006; Ducrotoy et al. 2017). *Brucella* transmission occurs primarily through reproductive fluids, aborted materials, and milk (Islam et al. 2023; Kiros, Asgedom & Abdi 2016). Following initial exposure to *B. abortus*, cows commonly abort once, while subsequent pregnancies are generally normal, although some may experience repeated abortions. Although non-pregnant cows and bulls are typically asymptomatic, they may still contribute to the maintenance and transmission of brucellosis within herds. Latent infections in heifers and vertical infections in calves further support disease persistence, while infected bulls may occasionally develop systemic illness (Neta et al. 2010; USDA 2022). A study in Southern India reported a high brucellosis prevalence among pigs, with 19 seropositive boars showing orchitis, suggesting that untested breeding males may have contributed to disease transmission within farms (Shome et al. 2019a). The transmission cycle associated with male animals, especially in breeding contexts, may be undervalued (Khurana et al. 2021). *Brucella*-infected bulls may have between 100 organisms/mL and 50 000 organisms/mL in their semen (Manthei, Detray & Goode 1950). These animals can spread the infection through both natural breeding and artificial insemination (Acha 2001; Khurana et al. 2021). In rams, brucellosis has been associated with reduced semen quality, including reduced total sperm output, poor motility, and a high percentage of morphological abnormalities (Cameron & Lauerman 1976). The reduced semen quality may affect the bacteriological examination of the specimen for the identification of *Brucella* spp. This highlights the importance of targeting females in surveillance programmes, while acknowledging the potential for males to act as silent carriers.

In South Africa, Trichard et al. (1982) reported a case of unilateral orchitis in a bull caused by *B. abortus* biotype 1. Serological testing detected positive antibody titres while cultures remained negative a month before the onset of clinical signs; subsequent cultures confirmed infection. This finding demonstrated that brucellosis in bulls can be detected serologically prior to the development of orchitis, emphasising both the diagnostic value of serology in males and their potential role as sources of infection before clinical manifestation. Similarly, a study in South Dakota investigated seven seropositive bison bulls aged 3–4 years, from which *B. abortus* biovar 1 was isolated in six animals (Rhyan et al. 1997). One bull exhibited severe necrotising and pyogranulomatous orchitis, while four others presented with mild seminal vesiculitis. Routine herd screening typically focuses on females; however, evidence from published abattoir surveys (Djangwani et al. 2021; Kolo et al. 2019; Madzingira et al. 2023), case reports of orchitis (Rhyan et al. 1997; Trichard et al. 1982), and laboratory records from eradication programmes (Govindasamy et al. 2021) indicate that seropositive bulls also occur. In most of the studies mentioned, infected males were identified through slaughterhouse monitoring, movement, or export testing or follow-up investigations in herds showing clinical signs. Collectively, these studies illustrate that male animals can harbour and shed *Brucella* organisms even before overt clinical signs appear, underscoring their epidemiological importance in disease transmission.

Despite the introduction of the bovine brucellosis control scheme in South Africa in the 1980s, the disease continues to rise, particularly among livestock, due to limited resources, inadequate vaccination, poor husbandry, and unrestricted animal movement (De Boni et al. 2022). A recent study on brucellosis in South Africa reported a seroprevalence of 1.73% among male livestock (Mazwi et al. 2023). In addition, *Brucella* culture-positive cases (11/200; 5.5%) from slaughtered animals included three males, with the younger males testing seropositive on both RBT and CFT, while the older male was RBT-positive but CFT-negative (Kolo et al. 2019). These findings raise concerns about the persistence of brucellosis and the challenges of its potential eradication within herds. Similarly, in Namibia, a slaughterhouse-based survey reported four CFT-positive males among five CFT-positive animals overall (Madzingira et al. 2023). In South Africa, outbreaks of *B. melitensis* in sable antelope have repeatedly involved males, with bulls identified as index cases, testing seropositive, and yielding isolates from bull testes, underscoring their potential role in persistence of infection within herds (Glover et al. 2020). Serological testing, including RBT and CFT, is performed by accredited laboratories in South Africa for cattle and the African buffalo (*Syncerus caffer*). In addition, *B. melitensis* and *B. suis* serology is available but non-accredited tests, and no serological tests are permitted for wildlife unless accredited. This diagnostic gap impairs early detection and hampers effective outbreak containment.

Despite evidence of *Brucella* infection in males, there is limited documentation of seronegative yet infectious breeding males contributing to transmission in the field. This represents a critical gap in current surveillance strategies, which rely heavily on serological testing. This study documented brucellosis on two farms: a cattle farm in Gauteng and a sable antelope farm in the Limpopo province of South Africa. The study aimed to: (1) report brucellosis outbreaks in cattle and sable antelope herds, (2) evaluate the performance of serological and molecular diagnostics, and (3) assess the role of seronegative breeding males in disease transmission.

## Research methods and design

### Study design and sampling sites

**FIGURE 1:**
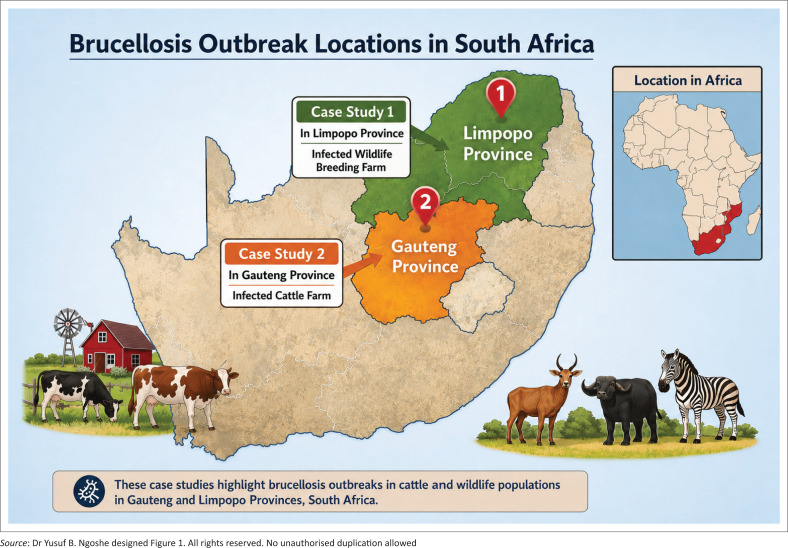
Map of South Africa showing the two provinces of the case studies reported.

This case study investigated the presence of *Brucella* spp. in two farms located in South Africa (Gauteng and Limpopo Province), with reported abortion events and suspected introduction of *Brucella*-infected bulls (Figure 1). The two cases involved:

**Case Study 1:** A cattle farm in Gauteng Province under quarantine because of historical *Brucella* infections detected since August 2024. Following a new abortion event when the person handling the aborted foetus tested positive for brucellosis, blood samples were collected from a newly introduced bull and 13 heifers housed separately to minimise within-herd transmission. The sample collection was carried out in October 2024. Following sample collection, the heifers were vaccinated with RB51 under the supervision of the state veterinarian. Blood samples were collected in yellow-top blood tubes and submitted to an accredited laboratory and to the Department of Veterinary Tropical Diseases (DVTD), University of Pretoria (UP), for serology and polymerase chain reaction (PCR).

**Case Study 2:** A wildlife breeding farm in Limpopo Province had documented brucellosis-infected African buffalo since 2016, with no reported abortions, although seropositive animals remained confined to their designated camp. Despite the separation of species into different camps, the farm reported abortion cases in the sable antelope herd housed in an adjacent camp to the buffalo in 2024. The herd was tested using serological tests, and two female sable antelopes were reported seropositive on RBT and CFT by an accredited laboratory. Samples were collected in September 2024 from a bull and two female sable antelopes with abortion histories. Specimens included semen and serum from the bull, as well as serum, lymph nodes, foetus, amniotic sac, and amniotic fluid from the brucellosis seropositive females upon culling. The samples were submitted to an accredited laboratory and DVTD for serology testing, culture, and PCR.

### Serological testing

#### Rose Bengal test

The RBT was conducted at the South African National Accreditation System (SANAS)-accredited serology laboratory and Biosafety Level 2 (BSL-2) laboratory of the DVTD, UP, according to the protocol.

#### Complement fixation test

The CFT was conducted at a SANAS-accredited serology laboratory in accordance with the WOAH protocol on RBT-positive samples with positive titres ≥ 30 CFT international units (IU)/ml for unvaccinated animals.

#### Indirect enzyme-linked immunosorbent assay (iELISA)

All positive samples from the RBT were confirmed by iELISA. The iELISA is both a sensitive and specific test (Legesse et al. 2023), which detects a broader range of immunoglobulin G (IgG) than RBT and CFT, including IgG2, and thus exhibits higher sensitivity for chronic infections (Corbel 2006; Khurana et al. 2021; WOAH 2023). The iELISA was performed using the ID Screen^®^ Brucellosis Serum Indirect Multi-species kit (Innovative Diagnostics, Grabels, France), following the manufacturer’s instructions. To minimise variability, each sample was tested in duplicate. Optical densities were measured using a BioTek enzyme-linked immunosorbent assay (ELISA) reader (Agilent Technologies, Santa Clara, CA, United States) The test was performed according to the manufacturer’s protocol, with the interpretation of the sample-to-positive (S/P) ratio values: S/P% ≤ 110% – Negative; 110% < S/P% < 120% – Doubtful; and S/P% ≥ 120% – Positive.

### Bacteriological examination

According to the WOAH, culturing and identifying the bacterium remain the definitive (gold standard) method for diagnosing brucellosis. Tissue samples (lymph nodes, foetus, amniotic sac) and semen were cultured for *Brucella* spp. on Centro de Investigación y Tecnología Agroalimentaria (CITA) selective media supplemented with antibiotics to suppress the growth of competing bacteria, as described by Ledwaba et al. (2020) and incubated at 37 °C with 10% CO_2_ for 7–10 days in a BSL-2 laboratory. Suspected colonies were sub-cultured onto blood agar for further growth over 1 week. Isolation of *Brucella* spp. was limited by overgrowth of faster-growing contaminants.

### Genomic deoxyribonucleic acid (DNA) extraction

Genomic DNA was extracted from cattle blood clot samples, and the colonies were isolated from sable antelope tissues. DNA from bacterial colonies was extracted using the Invitrogen DNA extraction kit. For blood clot samples, the PureLink Genomic DNA Kit (Thermo Fisher Scientific, Waltham, MA, United States) was used following the blood-specific protocol with minor modifications: blood clots were incubated at 55°C overnight with proteinase K prior to DNA extraction.

### *Brucella* genus and species amplification assays

A multiplex Abortus-Melitensis-Ovis-Suis PCR (AMOS-PCR) assay was used to differentiate *Brucella* spp. by targeting species-specific IS*711* insertion sequences, following the general approach previously described in the literature. The reaction incorporated four forward primers, each selective for one *Brucella* spp. together with a single reverse primer complementary to the IS*711* element. The sequences of the forward primers were *B. abortus*: GAC GAA CGG AAT TTT TCC AAT CCC, *B. melitensis*: AAA TCG CGT CCT TGC TGG TCT GA, *B. ovis*: CGG GTT CTG GCA CCA TCG TCG GG, *B. suis*: GCG CGG TTT TCT GAA GGT GGT TCA The reverse IS*711* primer used for all reactions was: TGC CGA TCA CTT AAG GGC CTT CAT.

Each species-specific forward primer was added to the reaction mixture at a working concentration of 0.1 µM, and the IS*711* reverse primer was included at 0.2 µM. Conventional PCR (cPCR) amplification was carried out with an initial denaturation at 95 °C for 5 min, followed by 35 cycles of denaturation at 95 °C for 1 min, annealing at 55.5 °C for 2 min, and extension at 72 °C for 2 min. A final extension step was performed at 72 °C for 10 min. Amplicons were resolved on agarose gels to determine species identity based on expected fragment sizes.

For the IS*711* real-time PCR (qPCR), the *B. abortus* BRuAB2_0168 and *B. melitensis* BMEII0466 primers and probes, as described by Hinić et al. (2008), were used. The qPCR amplification was performed in a total reaction volume of 25 µL, containing 12.5 µL Quantabio PerfeCTa qPCR SuperMix Low-Rox reagent (Whitehead Scientific, Cape Town, South Africa), 2 µL DNA, following the conditions suggested in Hinić et al. (2008), but using a 2-step amplification with the S19 vaccine as a control. The qPCR cycling conditions consisted of an initial denaturation at 95 °C for 3 min, followed by 40 cycles of denaturation at 95 °C for 30 s, annealing at 60 °C for 30 s, and extension. qPCR results with a cycle threshold (Ct) value of < 35 were considered positive for *Brucella* spp.

### Data sources and analysis

Animal identification was based on ear tags provided by farm owners. Epidemiological data were gathered through structured communication with farmers and provincial state veterinarians. All results were recorded and analysed using Microsoft Excel spreadsheet (Redmond, WA, United States), with preliminary results organized in a Microsoft Word document (Redmond, WA, United States).

### Ethical considerations

Ethical clearance to conduct this study was obtained as part of the research project, which was granted by the UP (REC046-24 and 439/2024). Section 20 approval for animal disease research was obtained from the Directorate of Animal Health, Department of Agriculture, Land Reform and Rural Development (DALRRD) for both the cattle and wildlife samples (Ref 12/11/1/1/5(6559OON) – cattle, 12/1/1/1/8(16912JPC), and 12/11/1/1/MG (1512) for the sable antelope).

## Results

### Case reports

#### Case study 1

According to the cattle farm’s June 2022 brucellosis testing report, the herd was declared brucellosis-negative following serological screening of 313 serum samples using the RBT (Table 1). Abortion cases were subsequently observed in 2024, and the local state veterinarian was notified by the farm owners.

**TABLE 1:** A summary of brucellosis testing history on the Gauteng cattle farm (June 2022 – January 2025).

Date	Test type(s)	Number of animals rested (male, female)	RBT positive	CFT-positive	Notes and/or outcome	Interventions
June 2022	RBT	313	0	0	Herd declared brucellosis-negative	No interventions
August 2024	RBT, CFT	2 (IDs 17012, 1901 females)	2	2	2 animals reported brucellosis positive	The herd was quarantined, and the 2 positive cattle were slaughtered
September 2024	RBT, CFT	8 (females)	8	7	Herd declared brucellosis positive	The herd remained quarantined
October 2024	RBT, iELISA, CFT, AMOS-PCR	14 (13 heifers + 1 bull)	14	13 (bull CFT 21)	Herd remained brucellosis positive	The herd remained quarantined
January 2025	RBT, CFT	10 (9 females) + 1 bull)	9 (bull negative)	7 (females)	Herd remained brucellosis positive	The herd remained quarantined

RBT, Rose Bengal test; iELISA, indirect enzyme-Linked immunosorbent assay; CFT, complement fixation test; AMOS-PCR, Abortus-Melitensis-Ovis-Suis Polymerase Chain Reaction.

In August 2024, two cattle (IDs 17012 and 1901) tested positive for brucellosis using RBT and CFT, with titres of 688 and 784 U/mL, respectively. Both animals were subsequently slaughtered. A young male residing on the farm, who was exposed to the infected animals and aborted materials, tested positive for brucellosis after handling an aborted foetus.

In September 2024, eight additional cattle (IDs 17032, 2202, 2008, 01, 2204, 05, 2212, and 2213) tested positive on RBT, while only seven were confirmed positive on CFT, with titres ranging from 72 U/mL to 784 U/mL. Cattle 17032 tested negative on CFT.

The UP’s investigation of the brucellosis-infected farm included a site visit in October 2024, during which 14 samples were collected from 13 heifers and one bull (Table 2). This follow-up visit was prompted by seropositive results detected in adult animals in September 2024. The bull, introduced for breeding purposes in 2020/2021, had an unknown brucellosis status at the time of introduction. Clinical examination during the October visit revealed no visible signs of illness in either the bull or the heifers. A human brucellosis case occurred following direct exposure to an aborted foetus on the infected cattle farm.

**TABLE 2:** *Brucella* serological and molecular test results from heifers and bull (October 2024).

ID	RBT	iELISA (SP value)	CFT (uL/mL)	Blood clots (AMOS-cPCR)
01	+	207.2243346	784	*B. abortus*
05	+	213.3586819	784	*B. abortus*
2004	+	213.3586819	784	*B. abortus*
2001	+	211.1787072	172	*B. abortus*
2002	+	213.7642586	784	*B. abortus*
2005	+	213.5361217	172	*B. abortus*
2008	+	212.1419518	784	*B. abortus*
2009	+	216.8060837	392	*B. abortus*
2012	+	203.599493	15	*B. abortus*
2013	+	204.6134347	86	*B. abortus*
2202	+	216.8567807	392	*B. abortus*
2208	+	213.9163498	72	*B. abortus*
17032	+	211.026616	581	*B. abortus*
Bull	+	ND	21	*B. abortus*

*B. abortus, Brucella abortus*; ND, Not determined; RBT, Rose Bengal test; iELISA, indirect enzyme-linked immunosorbent assay; CFT, complement fixation test; SP, sample-to-positive; AMOS cPCR, Abortus-Melitensis-Ovis-Suis Conventional Polymerase Chain Reaction.

Blood samples obtained from the 13 heifers and the bull were submitted for RBT and iELISA testing. All heifers, except heifer 2012, tested positive on RBT, CFT, and iELISA (Table 2). Heifer 2012 tested positive on RBT and iELISA and negative on CFT. The bull’s serum sample was submitted only to the Agricultural Research Council – Onderstepoort Veterinary Research for serological testing; therefore, no iELISA result was available for the bull. The bull tested positive on RBT and negative on CFT and was therefore classified as suspect. Subsequent testing consistently yielded negative RBT results, and the bull was thus considered seronegative for brucellosis.

DNA was extracted directly from blood clots using the PureLink genomic DNA kit (Thermo Fisher Scientific, Waltham, MA, United States). AMOS cPCR confirmed the presence of *B. abortus* DNA in all 14 animals tested (13 heifers and the bull) (Table 2).

In January 2025, the state veterinarian collected new blood samples for follow-up testing. The bull tested RBT negative, while nine cattle (17031, 02, 2003, 04, 17030, 17026, 2203, 2006, and 17001) remained RBT positive. Seven of these were confirmed positive by CFT (17031, 02, 04, 17030, 17026, 2006, and 17001).

During the same month, an abortion case was reported. A 6-month-old foetus was submitted to an accredited bacteriology laboratory for investigation. Tissue samples, including the lungs, liver, spleen, abomasal fluid, and umbilicus, were collected from the foetus. Based on culture identification, *Brucella* spp. was detected in the lungs, liver, spleen, and abomasal fluid, confirming the abortion as brucellosis-related.

#### Case study 2

The sable antelope (*Hippotragus niger*) herd investigated is in Limpopo Province, South Africa. The sable antelopes are kept on a farm near African buffalo that became infected with brucellosis in 2016, but were kept separately in a quarantine camp. No recent abortion cases were reported from the African buffaloes, but seropositive African buffaloes remained on the farm in the quarantine area, which is separate from the sable antelope camp. The original sable breeding herd was established in 2011 by purchasing animals from a private game breeder. The founding group consisted of one bull, five cows, and five calves (three heifers and two young bulls), with four of the cows already pregnant at the time of purchase. Since its establishment, bulls have been replaced approximately every 4 years to maintain breeding rotation and herd productivity (Table 3). The most recent bull (Bull 5) was introduced in June 2025 from a registered game breeder with unknown brucellosis testing.

**TABLE 3:** Epidemiological timeline of sable bull’s introduction on the farm (2011–2025).

Bull ID No.	Year introduced	Source	Remarks
Bull 1	2011	Initial herd purchased	Founding Bull
Bull 2	2014 (June)	Second bull purchased	Replaced the founding Bull
Bull 3	2018 (July)	Third bull purchased	Replaced Bull 2
Bull 4	2022 (March)	Fourth bull purchased	Replaced Bull 3
Bull 5	2025 (June)	Fifth bull purchased	Replaced Bull 4

In July 2024 and August 2024, a single female sable (ID: 20YE108) tested seropositive for *Brucella* antibodies during routine herd screening conducted by the state veterinary services. In response, the entire herd was retested in September 2024, during which two females (20YE108 and 22YE146) experienced abortions. Samples from the two females, including serum and tissues from the aborted foetus were submitted to DVTD for serology and molecular testing. Furthermore, a semen sample from Bull 4 was also sent to DVTD. The serum samples were confirmed positive for brucellosis using RBT, CFT, iELISA. *Brucella abortus* isolates were cultured from the lymph nodes and amniotic fluid from the two females (20YE108 and 22YE146). However, contamination prevented a pure culture, but it was confirmed by AMOS and qPCR as *B. abortus*, with qPCR Ct values of 27–32. Both animals were subsequently culled in accordance with provincial control measures. Notably, the sable antelope bull (Bull 4) yielded a negative RBT result but was subsequently confirmed *Brucella*-positive by bacterial culture of the semen sample (Table 4). The bull was not culled; instead, it remained in the herd and was replaced in June 2025.

**TABLE 4:** Serological, cultural, and molecular diagnostic results from sable antelope herd animals (2024).

ID	Tissues processed	Serum			Culture isolates	
		RBT	iELISA (SP value)	CFT (uL/mL)	*B. abortus* qPCR (Cq value)	AMOS cPCR
22YE146 (Female)	Lymph nodes	+	258.2216411	86	32.2	*B. abortus*
	Amniotic fluid		-	-	28.915	-
20YE108 (Female)	Lymph nodes	+	282.9682903	120	27.26	*B. abortus*
Bull 4	Semen	-	ND	ND	ND	*B. abortus*
Foetus tissues	-	-	-	-	-	Negative

ID, identification; *B. abortus, Brucella abortus*; RBT, Rose Bengal test; iELISA, indirect enzyme-linked immunosorbent assay; CFT, complement fixation test; qPCR, real-time polymerase chain reaction; ND, not determined; SP, sample-to-positive; Cq, quantification cycle; AMOS cPCR, Abortus-Melitensis-Ovis-Suis conventional polymerase chain reaction.

At the beginning of 2025, follow-up testing was performed on all remaining animals. One female (YE216) tested positive on serology. The animal was isolated and retested in June 2025, yielding a consistent positive result, after which she was also culled. As of mid-2025, the herd comprised 40 sable antelopes, distributed as follows: 1 bull, 17 cows, 12 heifers, and 10 young bulls with unconfirmed brucellosis status. Progressive herd testing indicated repeated detection of *Brucella*-positive females despite regular testing and replacement of breeding bulls.

**FIGURE 2:**
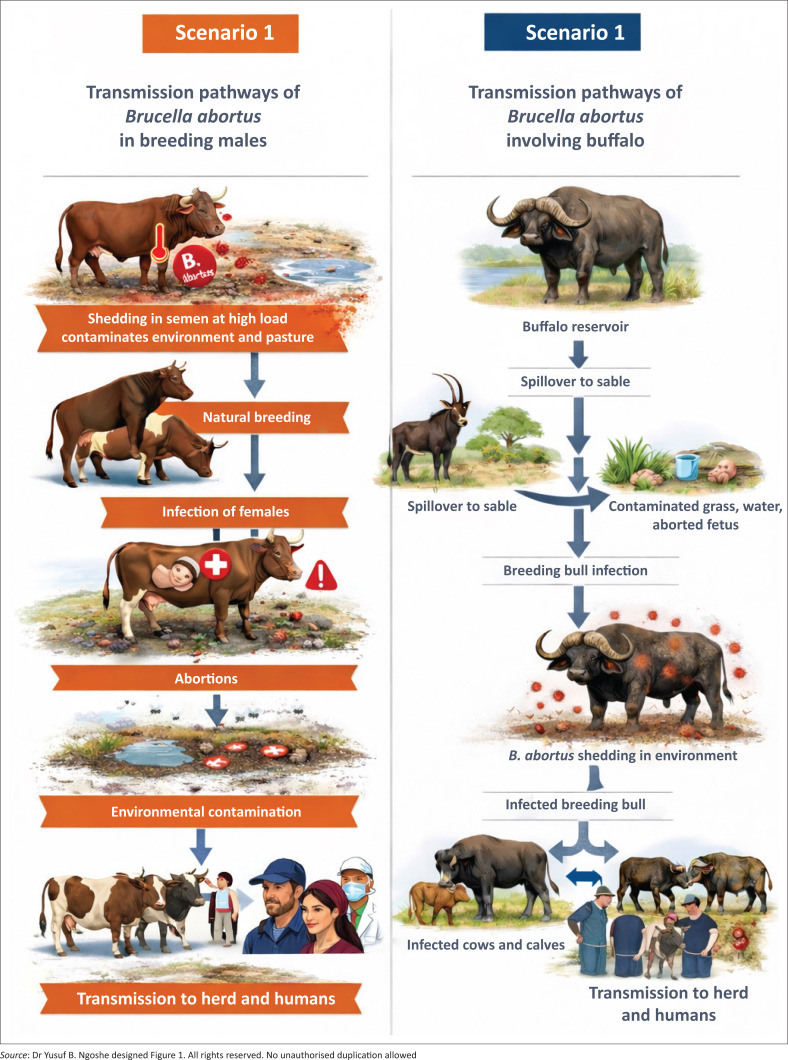
Possible transmission pathways of *Brucella abortus* in buffalo and breeding males.

Figure 2 presents two complementary transmission scenarios of *B. abortus* within livestock systems. Scenario 1 (Breeding males): This figure illustrates the possible transmission pathways of *B. abortus* originating from infected breeding males. Infection is maintained and amplified through infected breeding males that shed the pathogen in semen. During natural mating, this leads to infection of females, followed by reproductive losses such as abortions or shed semen in the environment, reported to be 100 organisms/mL – 50 000 organisms/mL (Manthei et al. 1950), which can contaminate the environment and pasture, leading to infection in the herd. These events contribute to environmental contamination, which further facilitates transmission to other herd members and potentially to humans.

Scenario 2 (Buffalo reservoir): Illustrates the transmission pathways of *B. abortus*. African buffalo act as a wildlife reservoir, with spillover occurring to susceptible species such as sable. Infection can then spread to breeding bulls and subsequently within the herd through direct contact and exposure to contaminated materials (e.g. aborted foetus, pasture, and water).

The comparison of diagnostic findings in the cattle and sable antelope cases demonstrates a consistent epidemiological pattern despite differences in host species, with *B. abortus* infection confirmed in both cases. In both herds, abortions served as the initial trigger for the outbreak, and seropositive females indicated active transmission within the population. However, a key distinction lies in the diagnostic profile of the breeding males: while the bull in the cattle herd showed suspect or variable serological results, the sable bull remained seronegative despite confirmed infection by culture/PCR (Table 4). In the cattle herd (caste study 1), the shift from a confirmed negative status in 2022 to the detection of seropositive cows in August–September 2024 indicates a likely recent introduction, with subsequent within-herd transmission (Table 5).

**TABLE 5:** Epidemiological timeline of infection.

Date	Farm status
**Case study 1**	
2022	Herd negative
August 2024	First positive cows
September 2024	Outbreak
October 2024	Bull tested
January 2025	Abortion confirmed *Brucella*
**Case study 2**	
2016	Buffalo infected
2024	Sable antelope abortion
2024	Females positive
2024	Bull semen culture positive
2025	Continued positive females

In contrast, the sable antelope outbreak (case study 2) appears to be linked to a long-standing environmental or wildlife source, which is likely associated with prior infection in African buffalo reported on the property in 2016. The occurrence of abortions and seropositive females in 2024, followed by the identification of a culture-positive breeding bull, suggests silent persistence and spillover of infection, with the male potentially contributing to amplification within the herd. Continued detection of positive females in 2025 supports ongoing transmission (Table 6).

**TABLE 6:** Comparison of diagnostic findings in cattle and sable antelope outbreaks.

Variables	Cattle herd (Case study 1)	Stable herd (Case study 2)
Species	Cattle	Sable antelope
Outbreak trigger	Abortions	Abortions
Female serology	Positive	Positive
Male serology	RBT suspect	RBT negative
Culture/PCR	Positive	Positive
Confirmed pathogen	*B. abortus*	*B. abortus*

PCR, polymerase chain reaction; RBT, Rose Bengal test; *B. abortus, Brucella abortus*.

## Discussion

Across both farms, brucellosis outbreaks were associated with the presence of infected breeding animals, particularly bulls, thus highlighting their role in herd-level transmission. On the Gauteng cattle farm, the transition from a brucellosis-negative status in 2022 to multiple abortion events and confirmed *B. abortus* infection in 2024 strongly suggests a recent introduction of the pathogen. Similarly, in the Limpopo sable antelope herd, routine screening in 2024 identified seropositive females, and *B. abortus* was confirmed by culture and molecular diagnostics. The detection of *B. abortus* in both female antelopes and a seronegative breeding bull indicates sustained transmission within the herd following initial spillover, consistent with evidence that breeding males may harbour and shed *Brucella* despite negative serology (Junqueira Junior et al. 2013; Khamesipour, Doosti & Taheri 2013; Rhyan et al. 1997).

Bulls infected with *Brucella* can shed the organism in semen, representing a recognised and biologically plausible route of venereal transmission during natural service (Acha & Szyfres 2001; Nicoletti 1990). Although the cow’s vaginal tract possesses natural defensive mechanisms, such as resident microbiota, local immune responses, and an acidic environment, these barriers do not completely prevent infection when *Brucella* organisms are introduced during mating (Quinn et al. 2011). *Brucella* is highly adapted to invade mucosal surfaces, including the genital tract, and even low numbers of organisms may penetrate the mucosa and establish infection (Corbel 2006). The risk is heightened in pregnant cows, as the gravid uterus contains erythritol and related polyols that support rapid bacterial replication (Jain et al. 2011). While venereal transmission is less common than exposure to aborted materials or contaminated environments, it remains epidemiologically relevant in brucellosis control programmes (WOAH 2023). In this study, infected breeding males, including seronegative individuals, likely contributed to within-herd transmission, posing a significant risk to the effectiveness of test-and-slaughter eradication strategies.

Despite repeated documentation of high *B. abortus* bacterial loads in the semen of infected bulls, natural venereal transmission is frequently described as inefficient or of limited epidemiological importance in the literature. Reported microbial concentrations ranging from 10^2^ to > 10^4^ organisms/mL of semen demonstrate clear biological plausibility for transmission during natural service; however, field observations seldom isolate transmission events solely attributed to mating (Acha 2001; Manthei et al. 1950; Nicoletti & Winter 1990). Several biological and epidemiological factors may explain this apparent discrepancy. During coitus, much of the ejaculate is expelled or lost from the reproductive tract (see Figure 2), and the bovine vaginal environment, characterised by resident microbiota, mucosal immune responses, and transiently acidic conditions, may reduce bacterial survival or limit ascending infection (Quinn et al. 2011). In addition, venereal exposure is typically brief and intermittent compared to prolonged environmental exposure to heavily contaminated aborted material, placentas, or uterine discharges, which are widely recognised as the dominant transmission pathways (Corbel 2006). These factors have contributed to a perception that venereal transmission plays only a secondary role in herd-level epidemiology, leading some surveillance frameworks to deprioritise routine testing of breeding males. However, this interpretation may underestimate the cumulative risk posed by infected bulls that service multiple females over extended breeding seasons. Even if the per-mating probability of transmission is low, repeated exposure events can substantially increase the likelihood of infection, particularly in pregnant females, whose gravid uterus provides a favourable environment for *Brucella* replication due to the presence of erythritol (Corbel 2006). Furthermore, venereal transmission may occur silently, without immediate clinical signs, delaying detection until abortions occur months later. From an epidemiological perspective, the limited emphasis on males in control programmes may therefore reflect diagnostic bias rather than true biological insignificance. Surveillance systems centred on abortion events and female serology inherently detect transmission only after amplification has occurred, while infected males, especially those that remain seronegative, escape early identification. Consequently, the contribution of breeding bulls may be systematically underestimated, reinforcing the view that venereal transmission is rare. The findings of this study challenge this assumption by demonstrating active infection in seronegative breeding males and providing field-based evidence that such animals may act as reservoirs, sustaining low-level, undetected transmission within herds over time.

The infection likely originated from a wildlife–livestock interface, given the historical presence of brucellosis-infected African buffalo on the farm, a transmission route repeatedly documented in Southern Africa’s multispecies systems, where buffalo are recognised maintenance hosts for *B. abortus* with the capacity to spill over to sympatric wildlife and livestock populations (Cossu et al. 2025; Ducrotoy et al. 2017; Godfroid et al. 2010). Although direct contact between buffalo and sable was not reported, indirect transmission through environmental contamination or shared interfaces cannot be excluded, consistent with previous reports demonstrating *Brucella* persistence in contaminated pastures, water resources, and fomites at wildlife-livestock interphases (Corbel 2006; Glover et al. 2020). The detection of *B. abortus* in both female antelopes and a seronegative breeding bull from which *Brucella* was isolated in semen indicates active infection and suggests sustained transmission within the herd following initial spillover. These findings align with published evidence in cattle, bison, and wildlife species showing evidence that breeding males may harbour and shed *Brucella* despite negative serology, thereby acting as reservoirs that are rarely detected through routine surveillance (Junqueira Junior et al. 2013; Khamesipour et al. 2013; Rhyan et al. 1997). Continued detection of seropositive females despite routine testing and bull replacement further supports ongoing transmission dynamics as previously described in endemic settings; here, undetected carriers contribute to long-term persistence even under test-and-slaughter strategies (Godfroid et al. 2010; Govindasamy et al. 2021). Collectively, these findings demonstrate that infected males can sustain and disseminate *Brucella* within herds, emphasising the need for rigorous, routine testing of breeding bulls in both livestock and wildlife systems, a recommendation increasingly supported by studies highlighting the epidemiological significance of male-mediated transmission and the limitation of female-centred surveillance frameworks (Khurana et al. 2021; WOAH 2023).

The diagnostic findings from both case studies highlight a critical limitation of serological testing, particularly in males. Several studies have shown that infected bulls often fail to mount detectable antibody responses, resulting in false-negative interpretations despite active infection (Corbel 2006; Junqueira Junior et al. 2013; Khamesipour et al. 2013). This phenomenon was evident in both the cattle and sable herds, in which *B. abortus* was detected in bulls by PCR and culture, respectively, despite negative or inconsistent serological results. Such animals represent silent carriers that may remain undetected during routine surveillance while continuing to shed the pathogen. From an epidemiological perspective, these undetected reservoirs complicate eradication efforts and necessitate the incorporation of complementary diagnostic approaches, including bacteriological culture and molecular assays. Bacteriological culture and molecular assays, particularly PCR targeting species-specific markers such as IS*711*, have been recommended to overcome the shortcomings of serology and improve detection in breeding males (Hinić et al. 2008; Legesse et al. 2023). In endemic areas, the use of serological tests such as RBT and CFT, detecting mainly IgM and IgG1, may fail to identify latent or chronic infections due to reduced sensitivity (Corbel 2006; Nielsen 2002; WOAH 2023). In contrast, the iELISA detects a broader range of IgG, including IgG2, exhibits higher sensitivity for chronic infections (Corbel 2006; Khurana et al. 2021; WOAH 2023). Complementary diagnostics such as bacteriological culture, PCR targeting species-specific markers (e.g. IS*711*), and iELISA, which detects IgG2 in addition to IgG1, are essential to overcome these shortcomings.

Within South Africa’s bovine brucellosis control scheme, new herds entering the programme must undergo two consecutive negative tests before a certified-area 3 (CA3) or brucellosis status declaration is issued by the state veterinarian. However, reliance on RBT and CFT alone may fail to detect all infected animals, particularly those with chronic or latent infection (Legesse et al. 2023; Shome et al. 2019b). The findings of this study demonstrate that breeding bulls not consistently identified as positive through serology may have contributed to bacterial transmission within the herd. Inter-herd transmission is commonly associated with the introduction of an infected animal (Radostits Otto et al. 2006), reinforcing the importance of pre-movement testing and biosecurity. These findings support the inclusion of iELISA and PCR-based assays as complementary diagnostic tools, particularly for detecting infection in animals that test negative on conventional serological tests. PCR assays targeting species-specific regions such as IS*711* further enhance detection by identifying *Brucella* DNA in blood and tissue samples (Ahmadi et al. 2021; Hinić et al. 2008; Legesse et al. 2023). This was demonstrated in this study by detecting *B. abortus* in blood clots from cattle and in semen and tissues from sable antelope.

*Brucella* infections in sable antelope have historically been associated with *B. melitensis*, including the identification of a sable-associated clade in South Africa (Mazwi et al. 2024). In contrast, this study reports *B. abortus* infection in sable antelope on a farm with a known history of *B. abortus* infection in African buffalo. Despite the absence of direct contact, indirect transmission through environmental contamination, fomites, or an unidentified intermediate host remains plausible. This finding underscores the need for enhanced biosecurity and systematic surveillance in multispecies game farms where *Brucella* can circulate silently across wildlife species. Overall, both timelines highlight delayed detection, the role of reproductive events as sentinel indicators, and the under-recognised contribution of breeding males in maintaining transmission, particularly in complex livestock–wildlife systems.

Currently, the bovine brucellosis control scheme in South Africa is voluntary, leading to limited compliance among livestock owners and inadequate enforcement of relevant legislation nationwide (De Boni et al. 2024). This study encourages the need for a national mandatory vaccination programme in livestock as a major control strategy for brucellosis in South Africa, where the infection is endemic. As reported by Zhang et al. (2018), to prevent and eradicate bovine brucellosis, breeding herds must have a vaccination rate of at least 80%. As of 2020, an overall vaccination of 36% was reported in Gauteng (De Boni et al. 2024), indicating that more vaccination programmes are required to achieve the recommended 80% vaccination rate. Vaccination lowers the incidence of abortions in livestock and will prevent the spillover to other hosts such as wildlife, prevent the spread of disease, and help protect herds from the illness. A coordinated national strategy that includes vaccination, movement control, mandatory bull testing, and farmer education is urgently needed.

Importantly, a confirmed case of human brucellosis in a young male, linked to exposure to an aborted foetus on the cattle farm, highlights the significant zoonotic risk associated with undetected infection in livestock. Human brucellosis remains underdiagnosed in South Africa, despite documented cases such as the first molecularly characterised infection reported in a 27-year-old male from the Western Cape Province (Wojno et al. 2016). Subsequent investigations in that case identified two additional human infections associated with goat handling, alongside a high proportion of seropositive animals in the affected herd, illustrating the close link between animal infection and human disease. Furthermore, genomic analysis has demonstrated close relatedness between *B. melitensis* isolates from humans and livestock in South Africa, supporting evidence of interspecies transmission (Mazwi et al. 2024). These findings, together with reports of human brucellosis across multiple African countries (Akakpo, Têko-Agbo & Koné 2009), underscore the importance of strengthening surveillance, improving diagnostic capacity, and increasing awareness among high-risk groups to reduce zoonotic transmission.

This study was limited by the small sample size, the inability to purify isolates for complete typing, and the reliance on blood clot DNA for PCR, which may reduce sensitivity. However, the findings provide critical evidence of hidden transmission risks, particularly the potential for seronegative male animals to infect females, as well as the shortcomings of current screening practices. In addition, heifers testing seronegative before sexual maturity and seronegative bulls that are unknowingly retained in breeding programmes pose a significant ongoing risk for perpetuating undetected transmission within the herd. Overall, the evidence demonstrates that infected males can sustain and disseminate *Brucella* within herds, reinforcing the need for routine bull testing and integrated diagnostics in South Africa.

## Conclusion

### Recommendations

This study demonstrates that breeding males may act as underrecognised reservoirs of *B. abortus*, with infection detectable even in seronegative animals. This finding highlights critical limitations of conventional serological testing and underscores the need to expand surveillance beyond female-focused strategies. The occurrence of a confirmed human brucellosis case following exposure to infected cattle further emphasises the zoonotic risk, the importance of targeted awareness campaigns, and the need for a One Health approach to disease control and prevention.

These findings underscore persistent challenges in brucellosis control in South Africa, including uncontrolled animal movement, inadequate vaccination coverage, and diagnostic blind spots in breeding males. The integration of molecular methods such as PCR alongside serological testing is recommended to improve detection, particularly in seronegative infected animals. Routine bull testing should be mandated to close the diagnostic gap and prevent silent transmission. In addition, a strengthened national vaccination strategy is critical to enhance herd immunity, reduce transmission, and support more effective control and eventual eradication of the disease. Coordinated measures combining vaccination, biosecurity, movement control, and farmer education will be essential to achieve sustainable progress.
